# Optical spin-symmetry breaking for high-efficiency directional helicity-multiplexed metaholograms

**DOI:** 10.1038/s41378-020-00226-x

**Published:** 2021-03-03

**Authors:** Muhammad Ashar Naveed, Muhammad Afnan Ansari, Inki Kim, Trevon Badloe, Joohoon Kim, Dong Kyo Oh, Kashif Riaz, Tauseef Tauqeer, Usman Younis, Murtaza Saleem, Muhammad Sabieh Anwar, Muhammad Zubair, Muhammad Qasim Mehmood, Junsuk Rho

**Affiliations:** 1 NanoTech Lab, Department of Electrical Engineering, Information Technology University (ITU) of the Punjab, Ferozepur Road, Lahore, 54600 Pakistan; 2 Department of Mechanical Engineering, Pohang University of Science and Technology (POSTECH), Pohang, 37673 Republic of Korea; 3 Department of Physics, Lahore University of Management Sciences (LUMS), Lahore, 54792 Pakistan; 4 Department of Chemical Engineering, Pohang University of Science and Technology (POSTECH), Pohang, 37673 Republic of Korea; 5 National Institute of Nanomaterials and Technology (NINT), Pohang, 37673 Republic of Korea

**Keywords:** Nanophotonics and plasmonics, Nanophotonics and plasmonics, Structural properties

## Abstract

Helicity-multiplexed metasurfaces based on symmetric spin–orbit interactions (SOIs) have practical limits because they cannot provide central-symmetric holographic imaging. Asymmetric SOIs can effectively address such limitations, with several exciting applications in various fields ranging from asymmetric data inscription in communications to dual side displays in smart mobile devices. Low-loss dielectric materials provide an excellent platform for realizing such exotic phenomena efficiently. In this paper, we demonstrate an asymmetric SOI-dependent transmission-type metasurface in the visible domain using hydrogenated amorphous silicon (a-Si:H) nanoresonators. The proposed design approach is equipped with an additional degree of freedom in designing bi-directional helicity-multiplexed metasurfaces by breaking the conventional limit imposed by the symmetric SOI in half employment of metasurfaces for one circular handedness. Two on-axis, distinct wavefronts are produced with high transmission efficiencies, demonstrating the concept of asymmetric wavefront generation in two antiparallel directions. Additionally, the CMOS compatibility of a-Si:H makes it a cost-effective alternative to gallium nitride (GaN) and titanium dioxide (TiO_2_) for visible light. The cost-effective fabrication and simplicity of the proposed design technique provide an excellent candidate for high-efficiency, multifunctional, and chip-integrated demonstration of various phenomena.

## Holography: Metasurfaces based on asymmetrical spin-orbit interactions (SOIs)

Metasurfaces are thin films composed of different elements that provide an efficient, miniaturized platform for nano-optics, and a metasurface based on asymmetrical SOIs has been developed that overcomes the limitations with metasurfaces using symmetrical SOIs. Owing to their ability to accommodate the wavefronts of light at the sub-wavelength scale, metasurfaces have found wide application in displays, communications, and data storage. However, metasurfaces based on symmetrical SOIs have limitations with holographic imaging. A team headed by Junsuk Rho at Pohang University of Science and Technology (POSTECH), South Korea has succeeded in designing a metasurface based on asymmetrical SOIs that operates in the visible light domain using low-loss dielectric materials. The team employed a simple, cost-effective fabrication method, and the design offers considerable potential for applications in such areas as asymmetric data inscription and smartphone displays.

## Introduction

Metasurfaces provide an efficient and miniaturized platform for nanophotonics due to their ability to tailor the wavefronts of light at the subwavelength scale. They have been applied in display applications^1,2^, communications^3^, beam engineering^4,5^, security^6^, and data storage^7^. Conventionally, in holography, computer-generated holography (CGH) algorithms are used to produce the required phase and amplitude profiles of a hologram^8^. In metasurface-based holography, plasmonic structures have been used in both reflection^9,10^ and transmission^11,12^. Li et al. proposed a multicolor metallic metasurface that can produce different images for the three primary colors, i.e., red, green, and blue. Crosstalk between kaleidoscopic holographic images is reduced using different optimal angles of incidence^13^. An ultrabroadband achromatic phase distribution is achieved by a catenary aperture-based plasmonic metasurface^14^. However, plasmonic metasurfaces that contain small metallic nanoresonators severely suffer from ohmic losses. As the ohmic losses lead to absorbance, the maximum theoretical efficiency decreases, while the measured efficiency is even lower due to fabrication defects^15,16^. This opens the door to exploring new materials to achieve highly efficient metasurfaces.

High-refractive-index dielectric materials are an ideal candidate for this purpose because they display strong electric and magnetic resonances in the visible domain^17–19^. Silicon is a widely used material for high-refractive-index dielectrics. Yang et al. proposed a polysilicon-based metasurface to achieve maximum cross-polarization reflection and the required phase at operational wavelengths of 1367–1380 nm^20^. Zhou et al. proposed a crystalline silicon-(c-Si)-based metasurface with 47% efficiency and full 0–2π phase control at the optimized working wavelength of 532 nm^21^. However, amorphous silicon (a-Si) exhibits very significant dielectric losses in the visible domain, which seriously reduces its efficiency^22^. Dielectric materials such as GaN and TiO_2_ are transparent in the visible domain with high-refractive indices. Ellipsometry data of GaN^23^ and TiO_2_^24^ strongly advocate their usage for fabricating efficient metalenses^25^ and metaholograms^26^. For example, a highly efficient and broadband metasurface using GaN nanopillars to produce wavefronts with arbitrary polarization based on given linearly polarized light has been presented^27^. Similarly, Khorasaninejad et al. demonstrated high-aspect-ratio TiO_2_ metalenses enabling diffraction-limited convergence at wavelengths of 660, 532, and 405 nm with transmission efficiencies of 66%, 73%, and 86%, respectively^28^. However, an incompatibility with existing CMOS technology is the major drawback of these material platforms. Moreover, the designs based on these materials possess high-aspect ratios (up to ~15) to achieve the required phase coverage, which leads to complex and costly fabrication processes^25,29^. To overcome these problems, a-Si:H-based metasurfaces have been proposed^30,31^. The addition of hydrogen to noncrystalline silicon lowers the absorption coefficient while maintaining a high-refractive index, comparable to that of GaN and TiO_2_. Therefore, the proposed a-Si:H can deliver a CMOS-compatible platform for fabricating high-refractive-index dielectric metasurfaces. Other exciting dielectric materials, such as indium tin oxide (ITO) and silicon nitride (SiN), have also been reported as potential candidates for implementation of high-efficiency metasurfaces operating in the visible domain^32,33^. However, the refractive indices of ITO (~2)^32^ and SiN (~2)^34^ are lower than that of a-Si:H (~3.25); therefore, nanorods made of ITO and SiN must be taller than those made of a-Si:H to achieve the required full phase modulation. Although ITO and SiN are well-known CMOS-compatible materials, a-Si:H promises simpler fabrication with smaller aspect ratios to achieve a similar functionality at the working wavelength.

Metasurfaces have shown great flexibility towards effective scaling and integration for multiple applications. However, most metaholograms only perform a single operation, i.e., they have half-space operation^5,35^. One way to realize bifunctional metasurfaces is to employ chiral structures that can utilize asymmetric transmission (AT) to design polarization-sensitive bioperational metadevices^36–38^. Such chiral AT effects originate from the breaking of the mirror symmetry of the structures and have been observed in multilayer structures^39,40^. These structures are vulnerable to complex fabrication processes; any mismatch in subsequent layers drastically reduces their efficiency. Chen et al. proposed a bifunctional and bilayer metapolarizer that attained efficiencies of 17% and 15.5% in the backward and forward directions, respectively, at an operating wavelength of 735 nm^41^. Chen et al. presented 3D helical metallic nanoapertures to achieve a direction-multiplexed and polarization-sensitive metasurface that can generate two unique wavefronts in the forward and backward directions under circularly polarized incidence and linearly polarized incidence, respectively. The maximum measured transmission efficiency of this metasurface was 20% at a wavelength of 800 nm^42^. Similarly, Chen et al. employed cascaded anisotropic impedance metallic sheets with different geometries to achieve asymmetric transmission, enabling direction-sensitive functionalities with a peak efficiency of 80% at the operation frequency of 8.6 GHz^43^. Zhang et al. demonstrated a single-layer spin–orbit-based metasurface exhibiting a very large AT in the mid-IR region^44^. However, the complexity of designing the proposed metasurfaces restricts scaling them down to the visible domain. Moreover, conventional metaholograms regulate the propagation of electromagnetic light by incorporating photon spin–orbit interactions based on the geometric phase. Such photon spin interaction-based metaholograms provide central-symmetric holography, which limits the helicity multiplexing approach. As a result, only 50% of the metadevice is active for a particular polarization handedness; therefore, the total efficiency of a geometric phase metadevice remains half regardless of its material properties.

**Fig. 1:**
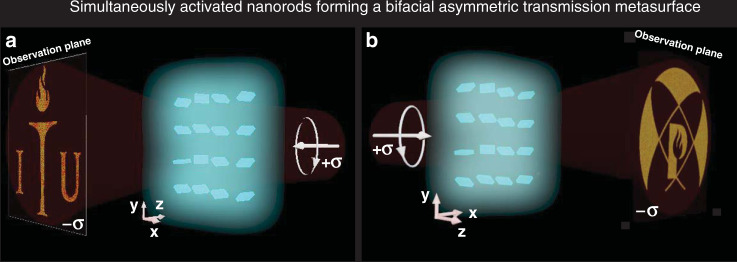
Operation of the proposed asymmetric wavefront generation metasurface. Right circularly polarized light incident from the **a** backward direction and **b** forward direction on a hydrogenated amorphous silicon (a-Si:H) metasurface. A single metasurface can produce two holographic images (the logo of POSTECH or Information Technology University “ITU”) depending on the propagation direction (see supplementary video part c).

Here, we propose an all-dielectric single-layer metasurface that enables asymmetric wavefront generation (AWG) for backward and forward incident circularly polarized light using a-Si:H nanoresonators. The phase-varying anisotropic antennas are spatially arranged for AWG such that the absence of mirror symmetry is maintained. The Pancharatnam–Berry (PB) phase originates from the rotation of the nanoresonators, and its value is reversed for the same circularly polarized light incident from different directions, while the retardation phase is direction-insensitive and depends on the physical dimensions and material properties of the nanoresonators^44^. In our proposed all-dielectric metasurface, both the PB and retardation phases are merged such that two independent and arbitrary pieces of information can be multiplexed into one metasurface, engaging the entire metasurface (unlike traditional geometric or PB phase metasurfaces). This proposed metahologram yields two holographic images, one from each side (backward and forward), depending on the light illumination direction, as depicted in Fig. 1. To prove this concept, a metasurface producing two highly efficient holographic images (logos of ITU and POSTECH) for antiparallel propagating circularly polarized (CP) incident light is simulated and fabricated. This proposed multiplexing technique based on asymmetric spin–orbit interactions has great potential in fabricating bifunctional metadevices.

## Methods and results

### Design methodology

**Fig. 2:**
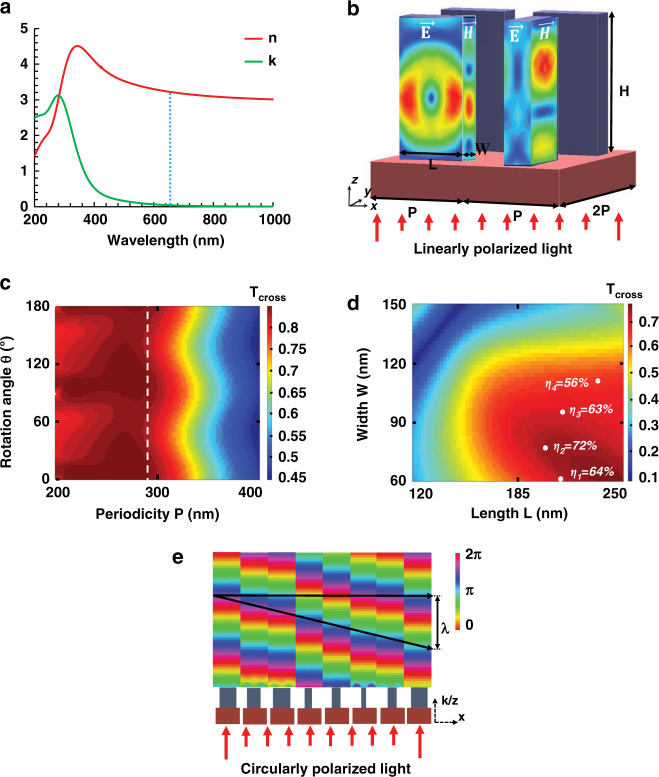
Optimization of the a-Si:H nanorods. **a** Measured values of refractive index (n) and extinction coefficient (k) of a-Si:H; at λ = 632.8 nm, the complex refractive index is 3.25 + 0.047j. **b** Unit-cell configuration composed of a-Si:H nanoresonators on a SiO_2_ substrate. The height (H = 400 nm) and period (P = 290 nm) are constant, but the length (L) and width (W) are different for all nanoresonators, depending on the required phase. For the two front nanoresonators, the $\vec E$ and $\vec H$ fields under x-polarized light, which are well confined in the nanoresonators, are depicted. **c** Efficiency of cross-polarization transmission (T_cross_) for a range of P and rotation angles (θ) of the a-Si:H unit cell. The white dashed line at P = 290 nm denotes where the average value of T_cross_ is ~0.97. **d** Efficiency of cross-polarization transmission (T_cross_) for different L and W. Four points are selected such that they have the maximum transmission efficiency (η) along with a mutual 45° phase difference (white dots are marked at the respective positions). **e** Wavefront of all nanoresonators for cross-polarized transmitted light at a working wavelength of 632.8 nm, obtained from full-wave simulations. 0–2π phase coverage and complete control over the wavefront of the transmitted light are realized.

High-refractive-index nanoresonators have a strong localized effect on shaping the phase and wavefront of incoming light. We can utilize an array of such nanoresonators to fabricate a metasurface, where each nanoresonator independently acts as a half-wave plate (HWP) or nanowaveguide^30,31,45^. a-Si:H nanoresonators are used on top of a silicon dioxide (SiO_2_) substrate, as illustrated in Fig. 2b. At the desired wavelength of 632.8 nm, the mobility gap and extinction coefficient of conventional a-Si are 1.7 and 0.47 eV, respectively. These values restrict its usage in the visible domain, as absorption becomes very prominent for wavelengths below 708 nm. With the inclusion of hydrogen, the mobility gap and extinction coefficient of a-Si can be increased and decreased, respectively, as the hydrogen eliminates the gap states and passivates the unstructured bonds^46^. The ellipsometry data depicted in Fig. 2a show that the measured refractive index of the proposed a-Si:H is 3.25 + 0.047j at 632.8 nm, which illustrates a significant decrease in loss compared to a-Si. The physical parameters (i.e., height (H), length (L), width (W), and periodicity (P)) of each unit cell are optimized to achieve the maximum cross-polarization transmission efficiency at the operational wavelength of 632.8 nm. The optimal periodicity while considering the Nyquist sampling criteria (P < λ/2NA, where λ and NA indicate the operating wavelength and numerical aperture) is achieved at 290 nm, as shown in Fig. 2c. The L and W of each nanoresonator are optimized using a parametric sweep while keeping P fixed at 290 nm. The cross-polarization transmission (T_cross_) efficiency for different L and W is shown in Fig. 2d. Four points are selected from the maximum T_cross_ and minimum T_co_ region (indicated by white dots) such that each nanoresonator has an incremental phase retardation of π/4 compared to its neighbor. The basic building block of our proposed metasurface is an array of such high-refractive-index nanoresonators. The H and P of each nanoresonator are kept the same, while the L and W are changed to produce the required phase profile. The optimized L and W for the four nanoresonators are as follows: 1 (W = 60 nm, L = 210 nm), 2 (W = 80 nm, L = 200 nm), 3 (W = 95 nm, L = 210 nm), and 4 (W = 110 nm, L = 230 nm).

**Fig. 3:**
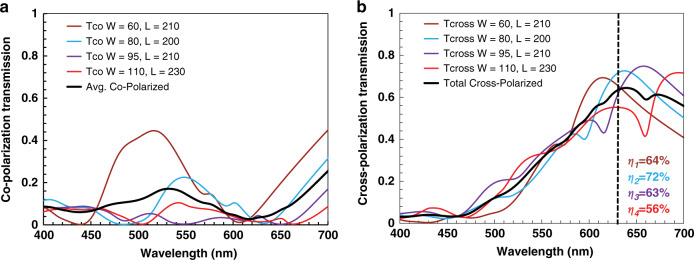
Broadband analysis of co-polarization and cross-polarization efficiencies. **a** Co-polarization components of all four optimized unit cells along with their average co-polarization. At the optimized wavelength of 632.8 nm, the average co-polarization transmission is minimized (≈6.2%). **b** Cross-polarization components of the four optimized unit cells along with their average cross-polarization transmission (η). At the operational wavelength, the average cross-polarization transmission is maximized (≈63%). The black dashed line indicates the working wavelength of 632.8 nm.

The broadband cross- and co-polarization transmission analyses of the optimized nanoresonators along with their average values are depicted in Fig. 3a, b. All of the nanoresonators have their minimum co-polarization transmission and maximum cross-polarization transmission (*η*) resonances at the optimized wavelength of 632.8 nm. This is evident from the average simulated value for cross-polarization transmission at a wavelength of 632.8 nm of ≈63%, while the average co-polarization transmission is limited to ≈6.2%. Higher cross-polarization efficiencies can also be acquired at wavelengths of 532 and 405 nm by utilizing a supercell scheme with unit elements individually optimized at three different wavelengths (632.8, 532, and 405 nm).

Resonance modes are studied for the individual nanoresonators. Both electric and magnetic resonance modes exist and are well confined within the nanoresonators. The a-Si:H nanoresonators show electric and magnetic resonance modes similar to those previously reported^47^, but with greater intensity due to the lower extinction coefficient in the visible domain. The dielectric resonance modes of the optimized nanoresonators with respect to photon spin interactions are depicted in Supplementary Fig. S1.

The total phase gradient of a nanoresonator is a combination of the PB phase and retardation phase. The PB phase originates from the rotational alignment of the nanoresonators, while the retardation phase depends on the material properties and physical parameters of the nanoresonators. By merging these two independent phases, we can achieve an asymmetric SOI^48^. Each nanoresonator acts as an HWP and imparts a phase shift of $\phi \pm 2\sigma \theta$ for the two orthogonal polarizations along its major and minor axes^44,48^. Here, *θ* defines the orientation angle of the nanoresonators with the *x*-axis, the sign ± represents the forward and backward propagation direction of the incident light, and $\sigma$ represents the circular helicity of the incident light. Moreover, *ϕ* denotes the value of the retardation phase. We have carefully designed and optimized four unit cells with an incremental phase difference of π/4 with respect to each other at the central operating wavelength of 632.8 nm. For full phase coverage from 0–2π, these four unit cells are repeated with a π/2 phase difference, as shown in Fig. 2d.

The retardation phase and PB phase can be independently controlled because these nanoresonators have low crosstalk due to the interresonator spacing being less than half of the working wavelength and hence act as weakly coupled Fabry–Pérot resonators^44,48^. Each nanoresonator simultaneously contributes to the forward and backward phase profile of the metasurface. The selection of the nanoresonators and their orientation relative to the *x*-axis can be performed using the following equations (the proof is given in supplementary information: Section 2):

1 $$\phi \left( {x,y} \right) = \frac{{\psi _b\left( {x,y} \right) + \psi _f(x,y)}}{2}$$

2 $$\theta \left( {x,y} \right) = \frac{{\psi _b\left( {x,y} \right) - \psi _f(x,y)}}{4}$$

Here, $\psi _b$and $\psi _f$ represent the required phase profile distributions for backward- and forward-propagating circularly polarized light, respectively. Eight quantized levels of the calculated phase are defined for appropriate selection of the nanoresonators, with each being placed at the corresponding position on the metasurface with a specific rotation *θ*.

### Experimental verification

For realization of a metasurface-based hologram, the phase profile required for the output beam needs to be determined. The Gerchberg–Saxton (GS) algorithm is the simplest technique that can provide the amplitude of the incident field along with the desired phase pattern in the far-field^49^. Using the modified GS algorithm, phase matrices for two separate logo images (ITU and POSTECH) were calculated separately. The phase matrix of one image (ITU) was selected for $\psi _f$, with that of the other image (POSTECH) being selected for $\psi _b$. These forward and backward phases were multiplexed according to Eqs. (1) and (2) for *ϕ* and *θ*. The calculated values of *ϕ* were subdivided into eight quantized levels ranging from 0–2π in ascending order. Each nanoresonator was employed at a corresponding position on the metasurface by comparing its phase with these quantized levels. The orientation of the nanoresonators was defined by the corresponding value of *θ*.

**Fig. 4:**
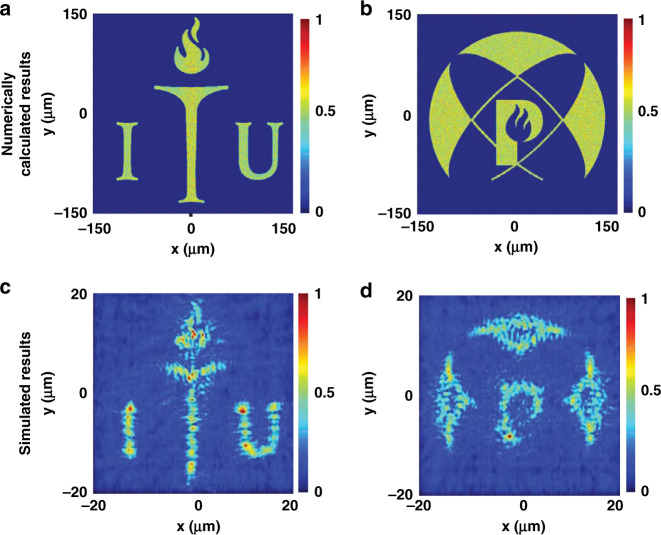
Numerically calculated and simulated results for the asymmetric wavefront generation metasurface. Numerically calculated phase map for an ≈300 × 300 μm^2^ metahologram for RCP light incident in the **a** forward and **b** backward directions. Simulated results for a 40 × 40 μm^2^ size Fourier-type metahologram obtained from finite-difference time-domain simulations (using FDTD Lumerical software) at a focal distance of 20 μm for RCP light incident in the **c** backward and **d** forward directions.

To verify the proposed concept, initially, a modified GS scheme was used to calculate a (1036 × 1036) phase matrix for the two logos, which corresponds to an ~300 × 300 μm^2^ asymmetric wavefront generation metasurface. The numerically calculated far-field images of these phase maps are depicted in Fig. 4a, b. Simulation results obtained by a finite-difference time-domain (FDTD)-based full-wave solver are depicted in Fig. 4c, d. The ITU logo appears when RCP light impinges on the metahologram from the backward direction, and the POSTECH logo appears under RCP illumination from the forward direction.

**Fig. 5:**
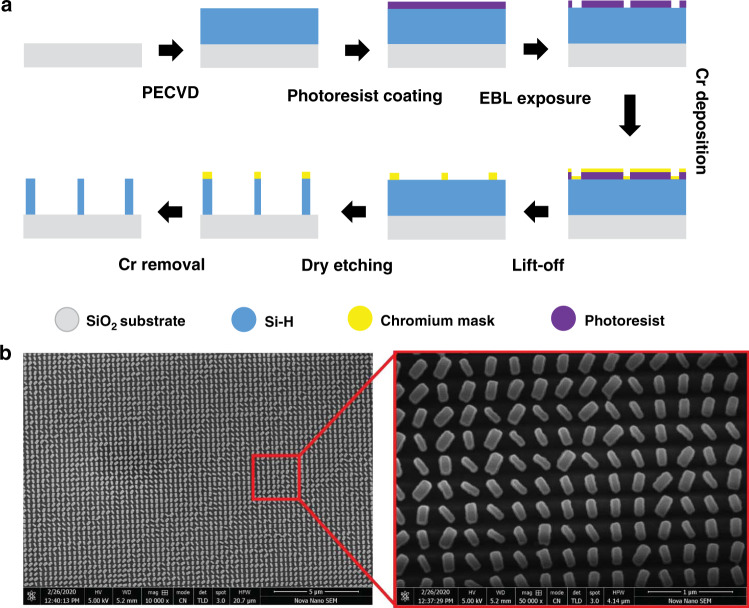
Fabrication steps and SEM images of the metasurface. **a** The metahologram was fabricated through the PECVD process. First, an a-Si:H layer was grown on top of a 500-μm thick SiO_2_ substrate. The deposition rate of a-Si:H was controlled by managing the flow rates of saline (SiH_4_) and hydrogen (H_2_) gases. The a-Si:H nanorods were defined by electron-beam lithography on a positive-type photoresist. A 30-nm thick chromium (Cr) layer was deposited followed by the lift-off process. The geometrical arrangement was transferred to a-Si:H using the Cr mask and dry etching. **b** SEM images of the designed asymmetric wavefront generation metasurface. The right image shows a magnified view of our fabricated hydrogenated noncrystalline silicon nanoresonators on top of a SiO_2_ substrate.

Plasma-enhanced chemical vapor deposition (PECVD) was utilized to fabricate an ≈300 × 300 μm^2^ asymmetric wavefront generation metahologram. A 400-nm thick layer of a-Si:H was grown on top of a 500-μm thick SiO_2_ substrate. The deposition rate of a-Si:H was maintained at 1.3 nms^−1^ at 300 °C by controlling the flow rates of silane (SiH_4_) and hydrogen (H_2_) gases at 10 and 75 sccm, respectively. Then, electron-beam lithography was employed to define rectangular-shaped a-Si:H nanoresonators on the patterned scanned positive photoresist. A 30-nm thick chromium (Cr) layer was deposited, followed by the lift-off process. The geometrical structures were transferred to the a-Si:H using the Cr mask and dry etching. A pictorial depiction of the fabrication steps for the asymmetric wavefront generation metaholograms along with final scanning electron microscope (SEM) images are presented in Fig. 5a, b.

**Fig. 6:**
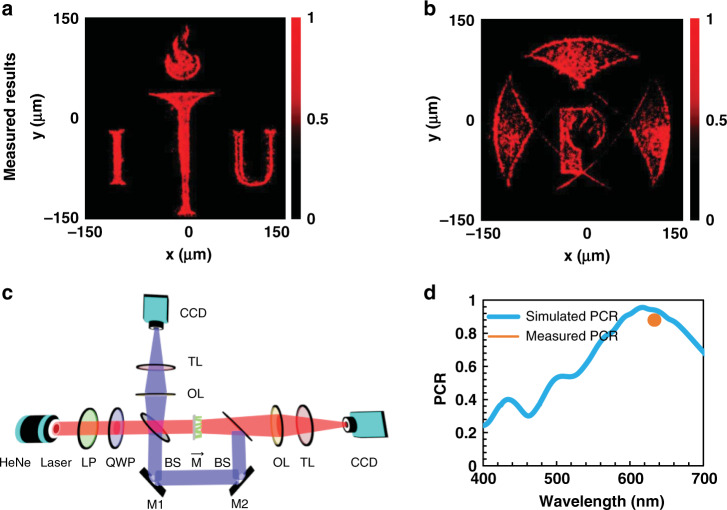
Optically measured results obtained from the asymmetric wavefront generation metasurface. Optically measured results obtained after characterization of the 300 × 300 μm^2^ metahologram, displaying the generation of two holographic images for RCP light incident in the **a** backward and **b** forward directions (see supplementary video part ‘b’). **c** Optical characterization setup used in the amplitude profile measurement of the metahologram^50^. **d** Polarization conversion ratio (PCR) of the proposed metasurface. At the designed wavelength of 632.8 nm, the simulated PCR is 91%.

The optical characterization results and corresponding setup used for the characterization of our fabricated AWG metahologram is depicted in Fig. 6a-d. A 5 mW HeNe laser was used as the incident light source at an operational wavelength of 632.8 nm. A quarter-wave plate (QWP) along with a linear polarizer (LP) was used to covert the light into LCP or RCP light. This circularly polarized light was passed through the beam splitter (BS) and divided into two parts with a 50/50 ratio. The metasurface sample was positioned next to the beam splitter on a two-dimensional stage at the desired beam waist. One part of this light passed through the metasurface in the forward direction and produced one holographic image (the logo of POSTECH) as shown in Fig. 6a. When mirrors M1 and M2 were active, the rest of the light was reflected through them (represented by the blue path). Another beam splitter was employed to impinge the light onto the metasurface from the opposite side (antiparallel to the first case). A second hologram (the logo of ITU) appeared in the backward direction as presented in Fig. 6b. Two charge-coupled device (CCD) cameras were used to capture the images through a ×40 objective lens (OL) and a tube lens (TL) on both sides as seen in Fig. 6c. In the complete characterization process, the sizes of both the incident and output optical beams were kept equal. The transmitted optical power was measured to obtain the transmission efficiency of the metahologram using a CCD camera. The incident power (P_in_) from the source was measured directly with the CCD camera, while the cross-polarized optical power (P_cros-pol_) was measured in the far-field of the metahologram. The total efficiency of the metahologram is calculated by the following formula [Eq. 3] (and measured efficiency at 632.8 nm wavelength is represented in Fig. 6d):

3 $$\eta = \frac{{{\mathrm{P}}_{{\mathrm{cros}} - {\mathrm{pol}}}}}{{{\mathrm{P}}_{{\mathrm{in}}}}}$$

The measured transmission efficiency for the designed asymmetric wavefront generation metahologram is 59%. The reconstructed images of the POSTECH and ITU logos projected in the far-field by the metahologram are depicted in Fig. 6a, b. Both images are very clear and have high fidelity at the incident wavelength of 632.8 nm. One possible reason for the slight blemish on the output in Fig. 6b is the imperfect conversion of the unpolarized incident light into circularly polarized light, which could be resolved using near-perfect polarization filters. The metahologram simultaneously provides a single image for forward and backward illumination. The polarization conversion ratio (PCR) is also a basic parameter to define how much incident light is converted into cross-polarized light. The PCR is described as the ratio of the cross-polarized component to the sum of the cross- and co-polarized components. The simulated average PCR for the proposed metasurface is 91%, depicted by the blue line in Fig. 6d, while the measured PCR is 88%, highlighted by the brown dot.

## Conclusion

In summary, a transmission-type multifunctional metasurface, which is CMOS process compatible and operates in the visible domain at a wavelength of 632.8 nm, has been demonstrated. A low-loss material, a-Si:H, with a high-refractive index and a low extinction coefficient for visible light was used, which relaxes the fabrication challenges faced when using other dielectric materials. Moreover, asymmetric spin–orbit interactions were manipulated such that they enabled asymmetric wavefront generation. The proposed technique controls the wavefront and independent phase modulation simultaneously by exploiting the whole metasurface array, in contrast to conventional symmetric helicity-multiplexed metasurfaces. We designed and fabricated a 300 × 300 µm^2^ metasurface to produce two central-symmetric holographic images of the POSTECH and ITU logos at a focal length of 150 µm. Under red light (632.8 nm) incidence, the measured transmission efficiency of our proposed metasurface was 59%. Therefore, a high transmission was attained in the visible spectrum using a-Si:H without compromising its performance. Furthermore, advanced metasurface design methodologies, such as wavelength-decoupled metasurface fabrication^51^, phase-intensity complex modulation^52^, chiro-optical metaholograms^53^, and complex-amplitude modulation by orbital angular momentum^54–56^, will enable multicolor and high-resolution bifacial metaholograms. Additionally, recently developed nanoimprinting-based scalable manufacturing techniques will further accelerate the mass production of metahologram devices^57,58^. We believe that this is a significant step towards the implementation of multifunctional, CMOS-compatible, and integrated photonic devices such as image sensors^59^. Also, once the metaholograms are further integrated with active platforms such as liquid crystals, real-time dynamic and high-resolution metaholographic displays will be realized^60^.

## Supplementary information

Supplementary Information
