# Supplementary material for: Optical spin-symmetry breaking for high-efficiency directional helicity-multiplexed metaholograms
Source: Microsyst Nanoeng. 2021 Mar 3;7:5. doi: 10.1038/s41378-020-00226-x (PMC8433315; doi:10.1038/s41378-020-00226-x)
Supplement: Supplementary file 1 — Supplementary Information [file 41378_2020_226_MOESM1_ESM.docx]

**Supplementary Information**

Optical spin-symmetry breaking for high-efficiency directional helicity-multiplexed metaholograms

Muhammad Ashar Naveed^1†^, Muhammad Afnan Ansari^1†^, Inki Kim^2†^, Trevon Badloe^2^, Juhoon Kim^2^, Dong Kyo Oh^2^, Kashif Riaz^1^, Tauseef Tauqeer^1^, Usman Younis,^1^ Murtaza Saleem^3^, Muhammad Sabieh Anwar^3^, Muhammad Zubair^1^*, Muhammad Qasim Mehmood^1^* and Junsuk Rho^2,4,5^*

^1^Department of Electrical Engineering, Information Technology University of the Punjab, Lahore 54600, Pakistan

^2^Department of Mechanical Engineering, Pohang University of Science and Technology (POSTECH), Pohang 37673, Republic of Korea

^3^Department of Physics, Lahore University of Management Sciences (LUMS), Lahore 54792, Pakistan

^4^Department of Chemical Engineering, Pohang University of Science and Technology (POSTECH), Pohang 37673, Republic of Korea

^5^National Institute of Nanomaterials and Technology (NINT), Pohang 37673, Republic of Korea

^†^These authors contributed equally to this work.

^*^Correspondence:

Junsuk Rho: [jsrho@postech.ac.kr](mailto:jsrho@postech.ac.kr), Muhammad Qasim Mehmood: [qasim.mehmood@iut.edu.pk](mailto:qasim.mehmood@iut.edu.pk), Muhammad Zubair: [muhammad.zubair@itu.edu.pk](mailto:muhammad.zubair@itu.edu.pk)

## Supplementary Note 1: Design principle

As nanostructures on a metasurface are minor compare to input wavelength, so we only consider the zero order propagation, all other orders are evanescent. If we neglect the losses, we can represent a uniaxial crystal whose fast axis is along x-axis by Jones matrix as

$$J=\left( \begin{matrix} exp(i\varphi) & 0 \\ 0 & exp(-i\varphi) \end{matrix} \right)$$

By considering the optical axis orientation as free space, Jones matrix will be transformed in coordinate dependent matrix as

$$T(r,\varphi)=M(r,\varphi)JM^{-1}(r,\varphi)$$

Position-dependent Jones matrix for transmission can be written as

$$T\left( r,\varphi\right)=Cos(\varphi)\left( \begin{matrix} 1 & 0 \\ 0 & 1 \end{matrix} \right)+iSin(\varphi)\left( \begin{matrix} Cos2\theta& Sin2\theta\\ Sin\theta& -Cos2\theta\end{matrix} \right)$$

M in the above equation is

$$M(r,\varphi)=\left( \begin{matrix} Cos\theta& Sin\theta\\ Sin\theta& -Cos\theta\end{matrix} \right)$$

Where ϕ is the retardation phase of wave plate, θ is the angle formed between the fast axes and the x-axis. Consider a circularly polarized wave is normally incident on a metasurface. This incident wave can be written as

$$E_{in}\left( r,\varphi\right)=E_{0}\left( r,\varphi\right)\boldsymbol{.}\left( \begin{matrix} 1 \\ i\sigma\end{matrix} \right)$$

Then, output beam would be

$$E_{out}\left( r,\varphi\right)=T\left( r,\varphi\right) E_{in}\left( r,\varphi\right)$$

This can be written as

$$E_{out}\left( r,\varphi\right)=E_{0}Cos(\varphi)\left( \begin{matrix} 1 \\ i\sigma\end{matrix} \right)+{iE}_{0}Sin(\varphi)\exp\left( i2\sigma\theta\right)\left( \begin{matrix} 1 \\ -i\sigma\end{matrix} \right)$$

According to *E_out_* when a CP beam is normally incident, it primarily scattered into two waves: one with same polarization as of incident beam without phase change, and second with opposite polarization and a spin-dependent phase change 2σθ.

As a metasurface consists of small size nano-resonators, where each nano-resonator acts like half wave plate (HWP). For simplicity, phase shift imparted by each nano-resonator can be written as $\varphi\pm2 \sigma\theta$ for antiparallel propagating CP light.

By writing this phase shift separately for forward and backward direction

$$\psi_{f}\left( x,y \right)= \varphi(x,y)-2\theta$$

$$\psi_{b}\left( x,y \right)= \varphi(x,y)+2\theta$$

After rearranging above two equations for $\varphi(x,y)$ and $\theta(x,y)$

$$\varphi(x,y)= \frac{\psi_{b}\left( x,y \right)+\psi_{f}\left( x,y \right)}{2}$$

$$\theta(x,y)= \frac{\psi_{b}\left( x,y \right)-\psi_{f}\left( x,y \right)}{4}$$

Above two equations clearly define all necessary conditions required for asymmetric wavefront generation (AWG). $\varphi(x,y)$ helps to choose a suitable size (phase and amplitude) of a nano-resonator while $\theta(x,y)$ helps to define its rotation in a particular position on a metasurface. Above mentioned two equations are used for designing AWG metasurfaces.

## Supplementary Section 2: Resonance modes inside nano-resonators

Dielectric resonance modes for all nano-resonators at operational wavelengths are depicted in Figure S1. Both electric and magnetic resonances exist and are well confined in the unit cells.


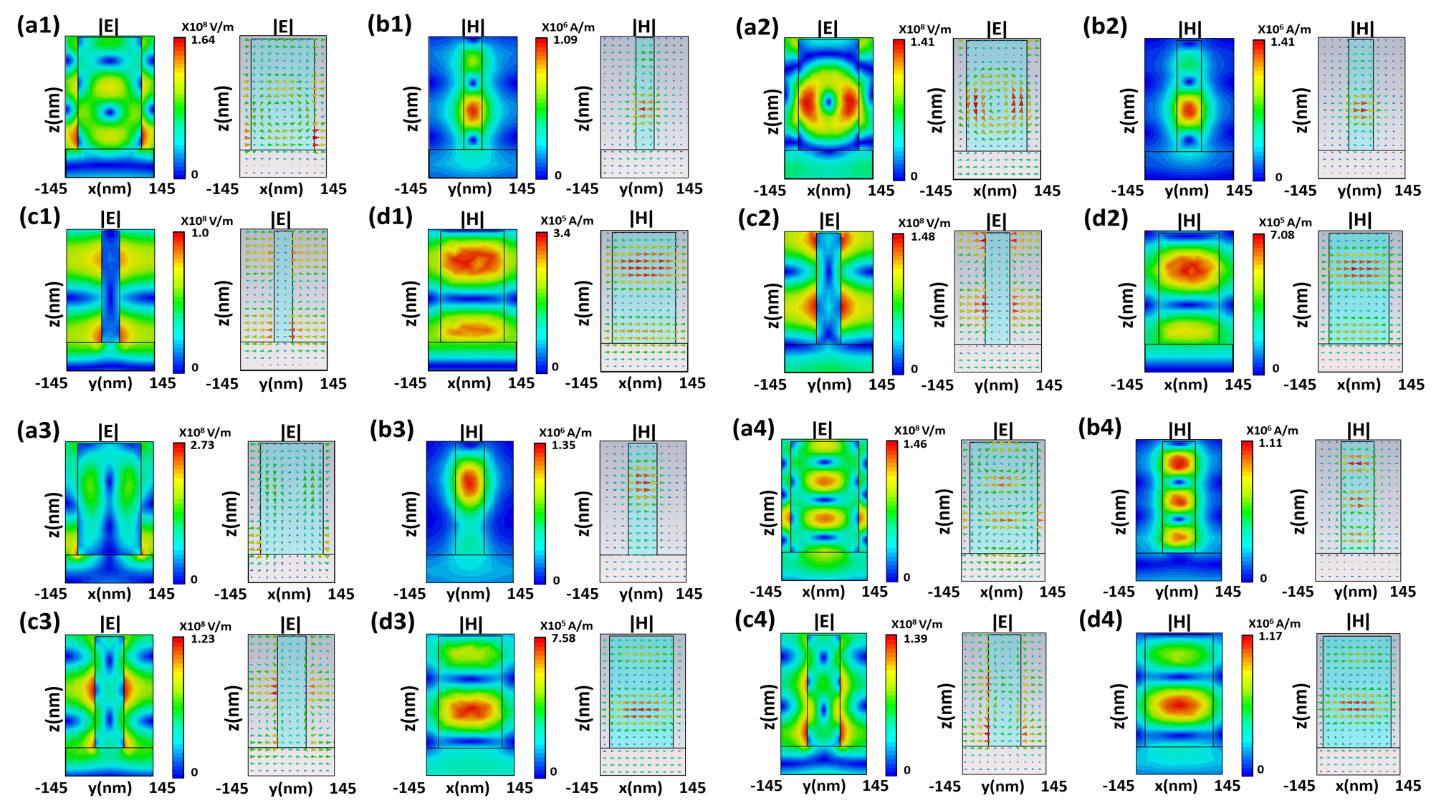


Figure S1. Resonance modes inside a-Si:H nano-resonators. Their placement order is: **(1)** a1 to d1 are for W=60 nm, L=200 nm **(2)** a2 to d2 are for W=80 nm, L=200 nm **(3)** a3 to d3 are for W=95 nm, L=210 nm **(4)** a4 to d4 are for W=110 nm, L=230 nm a, b) Cross-sectional views of field intensities and orientations under x-polarized incident light (a1 to a4) electric field (Vm^−1^) (b1 to b4) magnetic field (Am^−1^) c, d) Cross-sectional views of field intensities and orientations under y-polarized incident light (c1 to c4) electric field (Vm^−1^) (d1 to d4) magnetic field (Am^−1^). When x-polarized light is incident, an electric dipole is more intense as compare to a magnetic dipole, but both dipoles cramped to nano-rod. When y-polarized light is incident, a magnetic field is more dominant with a dipole resonance while an electric field shows quadruple resonance.

## Supplementary Section 3: Technology review:

Manuel Decker *et al.* presented a Huygens’ principle based metasurface which operates in near-infrared (NIR) region using silicon made nano-rods with 99% transmission efficiency.^[3]^ Wenyu Zhao *et al.* proposed a new advancement in the visible domain metasurfaces by designing a metahologram for three primary colors using silicon made nano-rods with a efficiency of 13.2%, 11.1% and 8.9% for red, green and blue light, respectively.^[4]^ Due to high extinction coefficients of silicon in visible domain, its usage remains very much limited to NIR region. Thus, this work enhanced the scope to explore novel materials and fabrication techniques to extend the operations of dielectric metasurfaces to the visible domain. Robert C. Devlin *et al.* proposed a highly efficient metahologram using an atomic layer deposition of TiO_2_ with overall efficiency greater than 78%. ^[5]^ Similarly, a GaN-based highly efficient metalens produced with the highest efficiency of 91.6% at working wavelength of 532 nm^[6]^. However, the fabrication methods of GaN- and TiO_2_-based metasurfaces are complicated due to their large aspect ratios (12 to 17.7) to achieve full phase coverage (0-2π). ^[5,6]^ Therefore, we have presented a metahologram using a-Si:H in transmission mode with a very high conversion efficiency of cross-polarized component (59%) in the visible domain with easy and straightforward manufacturability due to substantial reduction in terms of aspect ratio (up to 6.67). The proposed design methodology is highly suitable for practical applications and large scale production thanks to its cost-effectiveness and fabrication compatibility with mature processes in semiconductor industries.

| **Table S1** | | | | | |
| --- | --- | --- | --- | --- | --- |
| **Reference** | **Material** | **Max. Aspect Ratio** | **Device** | **Wavelength** | **Efficiency (%)** |
| Manuel Decker et al., Adv. Optical Mater. 2015^[3]^ | Si | 1 | Huygens’ Metasurface | NIR | 99 |
| Wenyu Zhao et al., Optics Letters. 2016^[4]^ | Si | 2 | Metahologram | Red, Green, Blue | 13.2, 11.1, 8.9 |
| Robert C. Devlin et al., PNAS. 2016^[5]^ | TiO_2_ | 15 | Metahologram | Red, Green, Blue | 78, 81, 82 |
| Bo Han Chen et al., Nano Letters. 2017^[6]^ | GaN | 12 | Metalens | Red, Blue, Green | 50, 91.6, 87 |
| Shuming Wang et al. Nature Nanotechnology. 2018^[7]^ | GaN | 17.7 | Achromatic Metalens | 400 to 660 nm | 61 |
| This work | Si-H | 6.67 | Metahologram | 633 nm | 59 |

## Supplementary references:

[1] Zhang, F., Pu, M., Li, X., Gao, P., Ma, X., Luo, J., Yu, H. & Luo, X. All‐Dielectric Metasurfaces for Simultaneous Giant Circular Asymmetric Transmission and Wavefront Shaping Based on Asymmetric Photonic Spin–Orbit Interactions. *Adv. Funct. Mater.* **27**, 1704295 (2017).

[2] Zhang, F., Pu, M., Luo, J., Yu, H. & Luo, X. Symmetry breaking of photonic spin-orbit interactions in metasurfaces. *Opto-Electron. Eng.* **44**, 319-325 (2017).

[3] Decker, M., Staude, I., Falkner, M., Dominguez, J., Neshev, D. N., Brener, I., Pertsch, T. & Kivshar, Y. S. High‐efficiency dielectric Huygens’ surfaces. *Adv. Opt. Mater.* **3**, 813-820 (2015).

[4] Zhao, W., Liu, B., Jiang, H., Song, J., Pei, Y. & Jiang, Y. Full-color hologram using spatial multiplexing of dielectric metasurface. *Opt. Lett.* **41**, 147-150 (2016).

[5] Devlin, R. C., Khorasaninejad, M., Chen, W. T., Oh, J. & Capasso, F. Broadband high-efficiency dielectric metasurfaces for the visible spectrum. *Proc. Natl. Acad. U.S.A.* **113**, 10473-10478 (2016).

[6] Chen, B. H., Wu, P. C., Su, V. C., Lai, Y. C., Chu, C. H., Lee, I. C., Chen, J.-W., Chen, Y. H., Lan, Y.-C., Kuan, C.-H. & Tsai, D. P. GaN metalens for pixel-level full-color routing at visible light. *Nano Lett.* **17**, 6345-6352 (2017).

[7] Wang, S., Wu, P. C., Su, V. C., Lai, Y. C., Chen, M. K., Kuo, H. Y., Chen, B. H., Chen, Y. H., Haung, T.-T., Wang, J.-H., Lin, R. M., Kuan, C.-H., Li, T., Wang, Z., Zhu, S. & Tsai, D. P. A broadband achromatic metalens in the visible. *Nat. Nanotechnol.* **13**, 227-232 (2018).
